# Reply to letter: Erosive balanitis caused by Staphylococcus haemolyticus in a healthy, circumcised adult male

**DOI:** 10.1099/acmi.0.000831

**Published:** 2024-06-04

**Authors:** José Mazuecos-Blanca, José Rafael Mazuecos-Gutiérrez, Ana Jiménez-Gil

**Affiliations:** 1 Dermatology Area, Department of Medicine, Faculty of Medicine, University of Seville, Sevilla, Spain; 2 Amate Health Centre, Seville District, Andalusian Health Service, Seville, Spain

**Keywords:** balanitis, balanoposthitis, circumcision, diabetes, lichen sclerosus

Sir,

The etiopathogenesis of lichen sclerosus (LS) is unknown and several factors are implicated. Chronic inflammation is one of the most reported [1]. The occlusive and moist environment under the foreskin may facilitate the development of male genital LS [2]. Chronic and intermittent exposure to urine, through urinary microincontinence, has been implicated [3], but also poor hygiene and retention of smegma [4]. A significant relationship has also been seen between patients with LS and diabetes [5], in addition to obesity and smoking [6].

Clinically, the typical involvement of male genital LS occurs on the glans and foreskin. Erythematous, shiny, and well-defined plaques evolving to whitish, indurated, and atrophic are observed. It is accompanied by pruritus, soreness, and tenderness, sometimes with dysuria [1]. When the sclerotic tissue formation progresses, the thickened foreskin cannot be retracted causing phimosis, with impairment of sexual intercourse with dyspareunia. The formation of painful, longitudinal fissures in the whitish sclerotic ring will hinder retraction of the foreskin and erection [2].

The treatment of choice for patients with LS and non-retractile foreskin is circumcision, which also allows to confirm the histological diagnosis. On the other hand, circumcision is also indicated in recurrent balanoposthitis, so balanitis is rare in circumcised patients and severe balanitis much more so [7].

Our patient did not report erythematous or whitish plaques on the glans penis, nor pruritus, pain, difficulty retracting the foreskin or dyspareunia in the years prior to genital surgery, suspicious for genital LS. No clinical manifestation of LS was also not observed after antimicrobial treatment, so this clinical image was not published as irrelevant. On the advice of his urologist, the patient underwent circumcision alone to prevent recurrent balanoposthitis [8].

However, we have also observed extensive balanitis with involvement of the glans and shaft of the penis caused by *Enterococcus faecalis* in a diabetic male two months after undergoing a postectomy for severe phimosis that prevented him from retracting the foreskin. The pathological study was diagnosed as lichen sclerosus. The patient reported not having sexual intercourse with his wife since the development of phimosis, so we thought that there was a previous genital colonization by this species of enterococcus [7].

In conclusion, although exploration of all possible diagnoses is beneficial and consideration of LS is reasonable, it does not appear that the patient with erosive *S. haemophilus* balanitis had a male genital LS, unlike the second patient with extensive *E. faecalis* balanitis, in whom both the sclerotic clinical manifestations and the pathologic study indicated a genital LS.

## Abbreviation

LS: lichen sclerosus
